# Immunological Involvement of MicroRNAs in the Key Events of Systemic Lupus Erythematosus

**DOI:** 10.3389/fimmu.2021.699684

**Published:** 2021-08-02

**Authors:** Mingxuan Chi, Kuai Ma, Yunlong Li, Min Quan, Zhongyu Han, Zhaolun Ding, Xin Liang, Qinxiu Zhang, Linjiang Song, Chi Liu

**Affiliations:** ^1^Reproductive & Women-Children Hospital, School of Medical and Life Sciences, Chengdu University of Traditional Chinese Medicine, Chengdu, China; ^2^Department of Nephrology, Osaka University, Suita, Japan; ^3^Department of Emergency Surgery, Shannxi Provincial People’s Hospital, Xi’an, China; ^4^Department of Nephrology, Sichuan Clinical Research Center for Kidney Disease, Sichuan Provincial People’s Hospital, University of Electronic Science and Technology, Chengdu, China

**Keywords:** microRNAs, systemic lupus erythematosus, epigenetics, autoimmune disease, DNA methylation

## Abstract

Systemic lupus erythematosus (SLE) is an archetype autoimmune disease characterized by a myriad of immunoregulatory abnormalities that drives injury to multiple tissues and organs. Due to the involvement of various immune cells, inflammatory cytokines, and related signaling pathways, researchers have spent a great deal of effort to clarify the complex etiology and pathogenesis of SLE. Nevertheless, current understanding of the pathogenesis of SLE is still in the early stages, and available nonspecific treatment options for SLE patients remain unsatisfactory. First discovered in 1993, microRNAs (miRNAs) are small RNA molecules that control the expression of 1/3 of human genes at the post-transcriptional level and play various roles in gene regulation. The aberrant expression of miRNAs in SLE patients has been intensively studied, and further studies have suggested that these miRNAs may be potentially relevant to abnormal immune responses and disease progression in SLE. The aim of this review was to summarize the specific miRNAs that have been observed aberrantly expressed in several important pathogenetic processes in SLE, such as DCs abnormalities, overactivation and autoantibody production of B cells, aberrant activation of CD4^+^ T cells, breakdown of immune tolerance, and abnormally increased production of inflammatory cytokines. Our summary highlights a novel perspective on the intricate regulatory network of SLE, which helps to enrich our understanding of this disorder and ignite future interest in evaluating the molecular regulation of miRNAs in autoimmunity SLE.

## Introduction

Systemic lupus erythematosus (SLE) is a severe autoimmune inflammatory disease with a broad range of clinical manifestations characterized by loss of tolerance to self-antigens, activation of dysregulated autoreactive T cells and B cells, production of autoantibodies (auto-Abs) and perturbed cytokine activities (1). Approximately 50% of SLE patients develop life-threatening complications, such as nephritis, vasculitis, pulmonary hypertension, interstitial lung disease, and cerebral stroke (2). Current studies suggest that SLE is associated with dysregulation of the innate and adaptive immune responses that are likely rooted in the intricate interactions among environmental stimulants, sex hormone imbalance, genetic predisposition, epigenetic regulation, immunological factors, and other undefined factors, resulting in breach of self-tolerance characterized by uncontrolled activation and expansion of dendritic cells (DCs) and lymphocytes, coupled with the production of copious amounts of anti-nuclear and antiphospholipid antibodies. Even so, current understanding of immunological events that trigger the onset of clinical manifestations of SLE is still in the early stages. At present, primary treatment for SLE is based on conventional nonspecific immunosuppressants, but this treatment option is unsatisfactory because of the associated side effects including infection, malignancy, metabolic disturbances, and infertility (1). Meanwhile, given the multitude of active pathways in a disease as heterogeneous as SLE (1), the role of single target approaches with inhibitors such as anti­CD20, anti­interferon­α and anti­IL­6 may be too limited. More extensive and in-depth study of SLE from different perspectives will contribute to a more comprehensive understanding of this disease and potentially open up exciting new therapeutic possibilities for treating this multifactorial disease.

MicroRNAs (miRNAs) are a large family of endogenous, single-stranded, small (~22 nucleotides), nonprotein-coding RNA molecules that modulate gene expression at the post-transcriptional level and protein synthesis in higher eukaryotes (3). In 1993, Lee et al. (4) firstly found that the *Lin-4* gene encoded some small RNAs, rather than proteins, which controlled the temporal development of *Caenorhabditis elegans*. With the development of molecular cloning and bioinformatics technology, more than 3,800 miRNAs have been identified so far, which are widely distributed in plants, animals, and viruses. Moreover, a recent study estimated that there are 2,300 mature miRNAs in humans (5), whose genes constitute about 1%–3% of the human genome sequence and approximately 1/3 of human genes expression is regulated by mature miRNAs (3, 6). It has been reported that the epigenetic ability of miRNAs can regulate a variety of biological processes, including embryo development, cell differentiation, proliferation, and apoptosis, signal transduction, and metabolism (7). In terms of the regulation of the immune system, increasing evidences suggested that miRNAs are involved in the regulation of innate and adaptive immune cells (8). It is not surprising that dysregulation of miRNA expression has been implicated in the progression of a broad range of diseases, some of which have been identified as diagnostic and/or prognostic biomarkers of various diseases, including cancer (9), diabetes (10), viral infection, cardiovascular diseases (11), and kidney diseases (12). In addition, miRNAs can affect the occurrence and development of autoimmune diseases through different pathways including the release of inflammatory mediators, innate immune responses, lymphocyte function, the signaling of toll-like receptors (TLRs), and nuclear factor (NF)-κB (13).

In 2007, Dai et al. (14) found differences in the miRNA expression profiles of SLE patients and normal controls, as seven miRNAs were down-regulated (miR-196a, miR-L7-5p, miR-409-3p, miR-141, miR-383, miR-112, and miR-184) and nine were up-regulated (miR-189, miR-61, miR-78, miR-21, miR-142-3p, miR-342, miR-299-3p, miR-198, and miR-298), suggesting that miRNAs are potential diagnostic markers of SLE and may be important factors related to the pathogenesis of the disease. With the publication of work of Dai et al. (14), more and more studies have demonstrated that the aberrantly expressed miRNAs have the ability to promote different immunological events of SLE, but the exact mechanism of these miRNAs is still largely unknown. The miRNAs in immune cells during active SLE has opened up potential new avenues for a more comprehensive understanding of SLE and may provide new therapeutic clues to improve patient outcomes, which remains to be confirmed and requires further investigation.

Alterations to miRNAs have been exploited as potential tools and targets for novel therapeutic approaches for many diseases. The first miRNA­based therapeutic agent was approved in 2013 for the treatment of familial hypercholesterolemia. Many miRNA-targeted therapies have been clinically advanced, including phase I clinical trials of miR-34, a mimic of the tumor suppressor miRNA for the treatment of cancer (15), and phase II clinical trials of anti-miRNAs targeting miR-122 for the treatment of hepatitis (NCT01646489, NCT01727934, NCT01872936, NCT01200420). MiRNAs have an intriguing potential role in the development and deterioration of SLE, which may allow for the development of more effective therapies with fewer side effects to mitigate this disorder. Therefore, this article reviews the current understanding of miRNAs and summarizes the impact of miRNA dysregulation in several important immunological events in SLE, including the dysfunction of immune-related cells, aberrant immune cell signaling, and the production of inflammatory cytokines. The summary of this study helps to enrich the current understanding of the intricate immunological regulation network of SLE and to stimulate future interest in evaluating the molecular regulation of SLE.

## Biogenesis and Function of miRNAs

**Figure 1:**
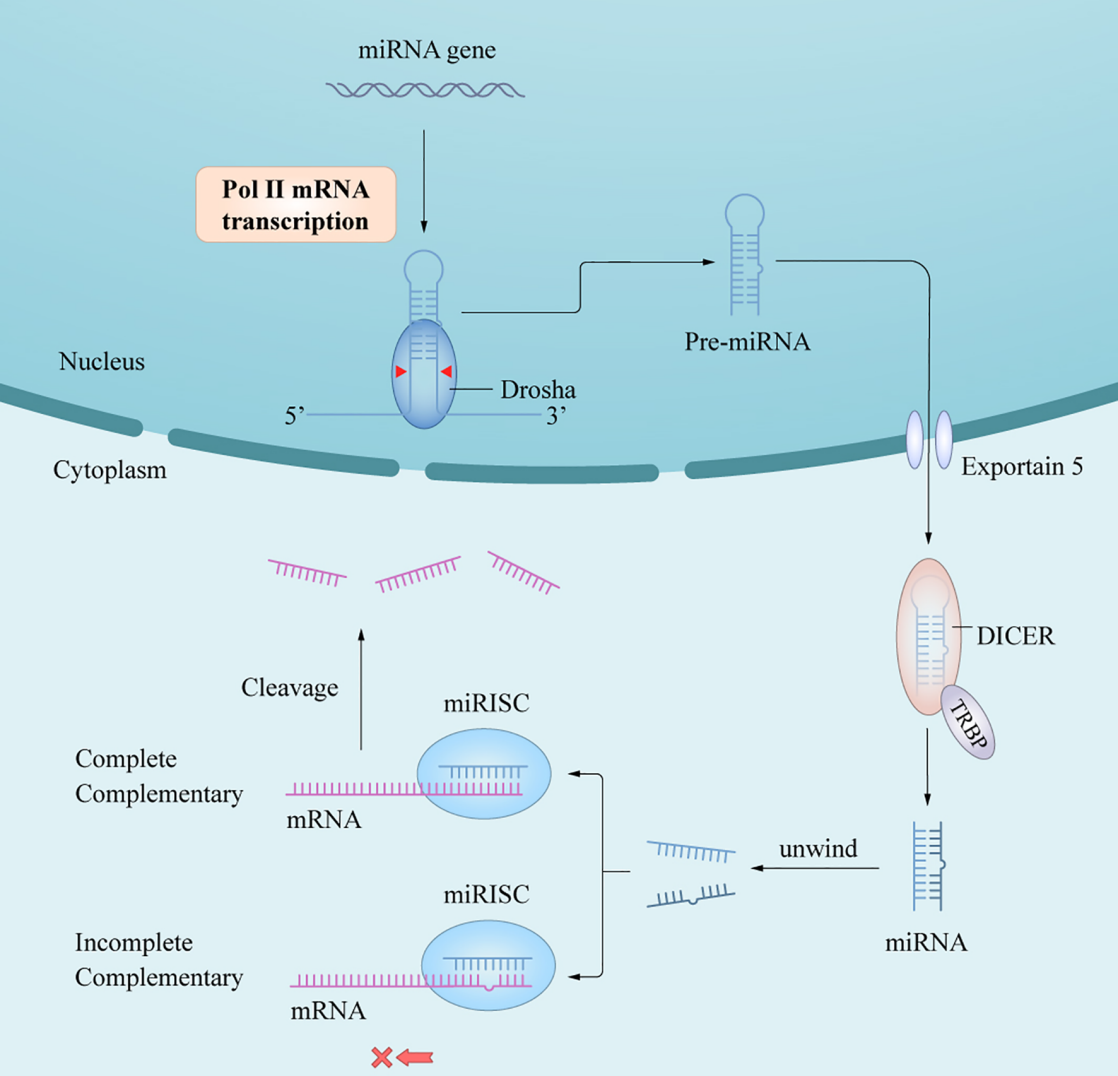
A *schematic of miRNA biogenesis.* miRNA biogenesis begins in the nucleus, where genes are mainly transcribed by RNA polymerase II into pri-miRNAs. Pri-miRNAs are processed into pre-miRNA by the RNase III enzyme, Dorsha and DGCR8. After being transported into cytoplasm by Exportin-5/Ran-GTP, pre-miRNAs are further processed by Dicer and TRBP to yield miRNAs duplex. An functional miRNA strand is unwound from the miRNAs duplex and then loaded into the RISC to form an miRISC assembly, which are the functional forms of miRNAs and the regulator of gene expression in posttranscriptional level by interacting with the target mRNA.

In humans and animals, synthesis of mature miRNAs is initiated by the transcription of nuclear genes into primary RNA transcripts (pri-miRNAs) (3), which are cleaved by the ribonuclease III (RNase III) enzyme Dorsha and the protein DiGeorge syndrome critical region 8 into precursor miRNAs (pre-miRNAs) (6). After being transported from the nucleus into the cytoplasm by exportin-5/Ran-GTP, pre-miRNAs are processed by Dicer and trans-activation response RNA-binding protein to yield miRNA duplexes. One of the functional chains is loaded into the RNA-induced silencing complex (RISC) to form an asymmetric RISC assembly (16), which interacts with the target messenger RNA (mRNA) to regulate the expression of target genes after transcription (17) (**Figure 1**) *via* two mechanisms of action depending on whether the single-stranded miRNA in the RISC assembly is completely complementary to the target mRNA 3’-untranslated region (3’-UTR); if so, the mRNA is targeted and degraded by the RISC assembly, and if not, the mRNA blocks translation of the target gene. The miRNAs mainly follow the first mechanism in plants and the second in animals. A complex regulatory network is formed between the miRNA and target gene mRNAs, thus affecting the course of disease through post-transcriptional regulation without changing the gene sequence (18). In short, the extent of complementarity between the miRNA seed region and the target mRNA 3’-UTR determines the mechanism of miRNA-mediated gene regulation/translation repression or miRNA cleavage and degradation.

## The Roles of miRNAs in DCs Abnormalities in SLE

**Figure 2:**
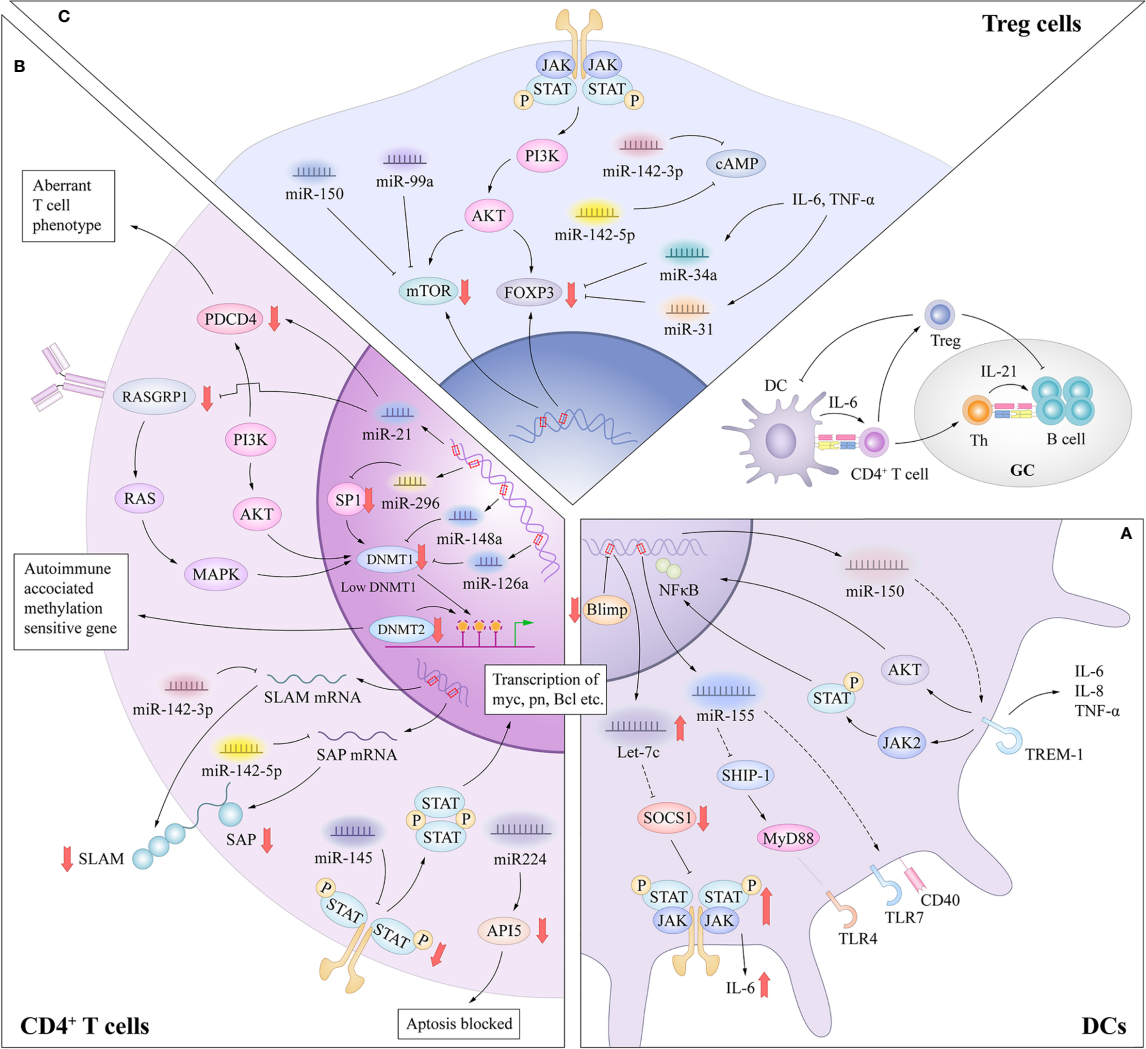
**(A)** MiRNAs in dysfunctional antigen presentation by DCs. In the DC-specific absence of Blimp1, an increase in let-7 miRNA results in a broad spectrum of proinflammatory DC phenotype, mediated in part through suppression of SOCS1 expression. The CD40 expression was significantly upregulated with a negative correlation to the miR-155 in SLE primary target SHIP-1 expression. MiRNA-150 inhibited the expression of TREM-1 which amplify the function of TLR4. **(B)** miRNAs in aberrant activation of CD4^+^ T cells. MiR-21 contributed to the aberrant phenotype of T cells through interaction with PDCD4 or indirectly inhibiting DNMT1 expression through targeting RASGRP1. miRNAs such as miR-126, miR-29b and miR-148a can directly inhibit DNMT1 expression by targeting the protein coding region. These processes result in the overexpression of autoimmune-associated methylation-sensitive genes, which contribute to the autoreactivity and overstimulation of CD4^+^ T cells in SLE. miR-142-3p specifically targets the SLAM family, while miR-142-5p targets the 3’-UTR of SAP. Thus, decreased miR-142-3p/5p expression contributes to the up-regulation of CD84 and IL-10/SAP, resulting in the increased T cell function and IgG production in co-cultured B cells. Aberrant expression of miR-145 and miR-224 can promote T cell activation-induced cellular apoptosis and SLE-associated nephritis by overexpression of STAT1 and underexpression of API5. **(C)** miRNAs in functional inhibitory of Treg cells. The release of IL-6 or TNF-α can increase the expression levels of miR-34a, which can attenuate Foxp3 expression by targeting its 3ʹ-UTR. MiR-142-5p positively regulates intracellular levels of cAMP to maintain the suppressive function of Treg cells. MiR-142-3p can restrict cAMP levels in CD4^+^ T cells, which compromises the inhibitory function of Treg cells. MiR-99a and miR150 could regulate the function of Treg cells by targeting mTOR.

In general, DCs have a unique sentinel function, continuously detecting danger signals from the environment through innate pattern-recognition receptors such as TLRs, which have the ability to capture antigens through binding to microbes or endogenous tissues (8). Inappropriate or dysfunctional antigen presentation by DCs might promote the breakdown of T­cell and B­cell tolerance in SLE (19). Patients with SLE show multiple DC abnormalities, including a decrease in the number of circulating normal myeloid DC (mDC) but an increase in the number of plasmacytoid DC (pDC). Similar to mDCs, pDCs upregulate the expressions of T-cell costimulatory molecules such as CD80, CD86 and CD40 upon antigen stimulation, and serve as antigen presenting cells to prime and activate T cells (20). Distinctively, the pDC subset specialize in producing type I interferon in response to single stranded RNA and hypomethylated CpG DNA, *via* TLR7 and TLR9 (21, 22). These unique features allow pDCs to play a crucial role in the pathogenesis of SLE and have been shown to correlate with disease manifestations including the SLE hallmark anti-dsDNA autoantibodies. Recent studies have described the involvement of let-7c, miR-155, miR-150 in regulating the functions of pDC in response to TLRs stimulation (**Figure 2A**).

B lymphocyte–induced maturation protein-1 (Blimp1) is identified as an important transcriptional repressor of let-7c miRNA (23). Expression of let-7c miRNA influences the differentiation and functional homeostasis in B cells and T cells respectively (24). In the DC-specific absence of Blimp1, an increase in let-7 miRNA results in a broad spectrum of proinflammatory DC phenotype, mediated in part through suppression of suppressor of cytokine signaling 1 (SOCS1) expression (23). According to this research, let-7c miRNA enrich our understanding of the mechanisms underlying of polymorphisms in Blimp1 associated with risk for human autoimmune disorders such as SLE and inflammatory bowel disease. The pDCs derived from symptomatic mice showed functional hypersensitivity to TLR7, as represented by the elevated upregulation of CD40, CD86 and MHC class II molecules. In addition, Yan et al. (25) showed an enhanced induction of miR-155 in SLE mice in response to TLR7 stimulation, and CD40 expression was significantly upregulated with a negative correlation to the miR-155 primary target SH2 domain-containing inositol 5’-phosphatase 1 (SHIP-1) expression. According to the research of Gao et al. (26), miRNA-150 inhibited the expression of TREM-1 which potently amplified the function of TLR4 and then enhanced the inflammation responses in splenic cDCs in lupus prone mice. These researches enrich our understanding of pathogenesis of DCs dysfunction in SLE. Compared with the role of miRNAs in adaptive immune cells, the contribution of miRNAs to DC activation has been examined in only a few studies, and further research is needed in this field.

## The Roles of miRNAs in Overactivation and Autoantibody Production of B Cells in SLE

**Figure 3:**
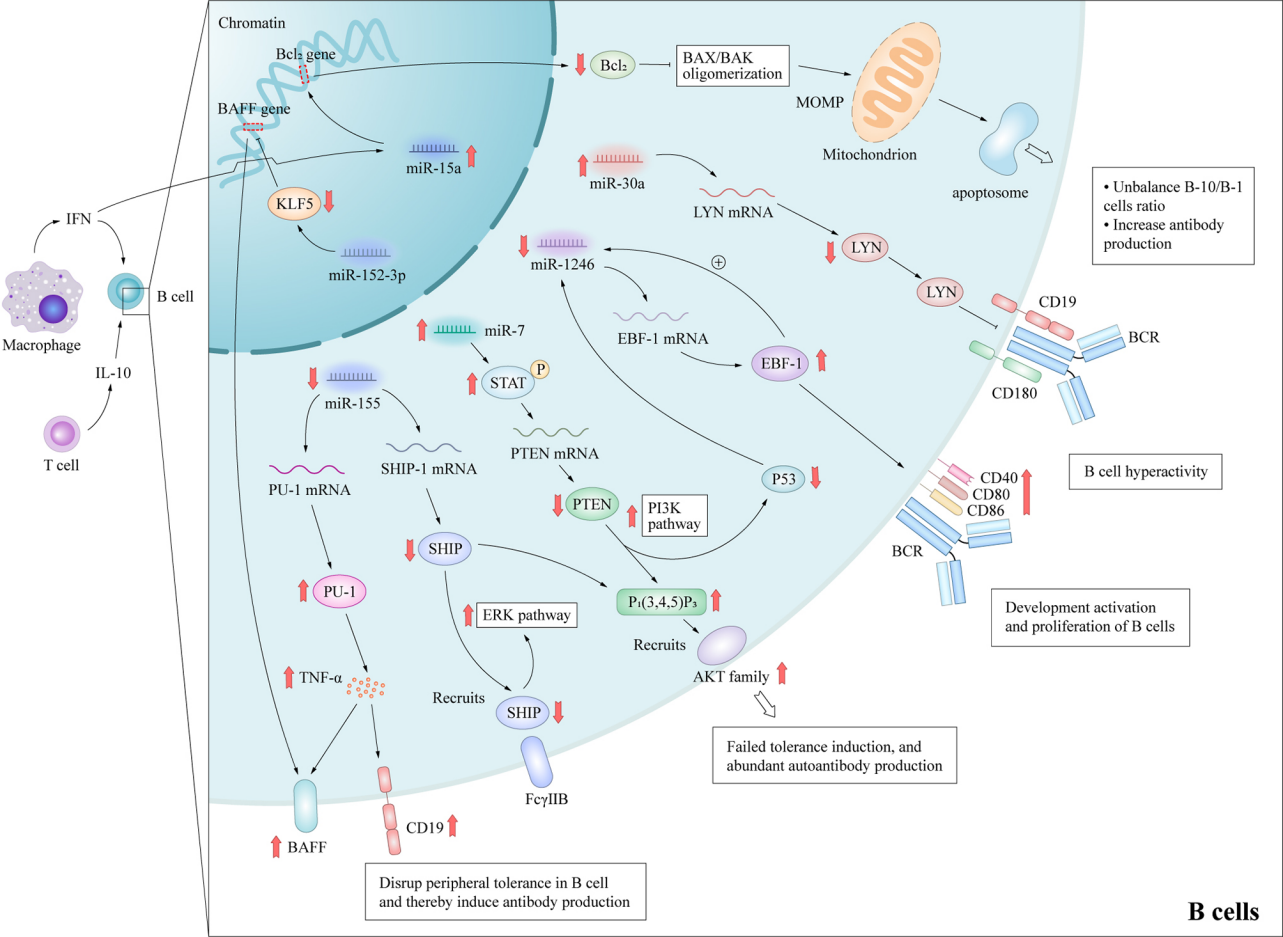
Molecular mechanisms by which miRNAs promote B cell hyperactivity in SLE patients. These aberrant expressed miRNAs promote B cells abnormal activation and autoantibodies production in SLE by affecting important protein molecules in the B cell signaling pathway. MiR-15a interacts the Bcl-2 gene directly, which leads to the decrease of Bcl-2 and activation of intrinsic apoptotic pathway of regulatory B-10 cells. MiR-7 mediates the suppression of PTEN/AKT signaling, and then promotes B cell differentiation into plasma cells and spontaneous germinal center formation. Decreasing miR-155 in B cells contributes to the SHIP-1 reduction, which leads to the production of serum IgG anti-dsDNA antibodies. MiR-155 also suppresses PU.1 and TNF-α, and then inhibits BAFF and CD19 protein expression, and promotes the B cell proliferation and antibody production in SLE. MiR-30a increases in B cells of SLE patients and directly decreases the expression of Lyn, an important mediator of B cell activation, *via* targeting with the 3’-UTR of Lyn mRNA. MOMP, Mitochondrial outer membrane permeabilization; BAX/BAK, Bcl-2-associated X protein/Bcl-2 homologous antagonist killer.

Abnormalities of B cells are important characteristics of the pathogenesis of SLE. Although it is well-known that B cells have the ability to produce autoantibodies, they also mediate venomous functions through antibody-independent activities, including the presentation of antigen to T cells, co-stimulatory functions *via* the expression of accessory molecules engaging stimulatory receptors on T cells and the production of cytokines (27). Furthermore, B cell depletion therapy can have beneficial effects on patients with these disorders (28), highlighting the importance of B cells in the pathogenesis of autoimmune diseases. These autoreactive antibodies promote the pathogenesis of through cause acute and chronic inflammation and tissue necrosis with the participation of complement, or cause tissue cell destruction by directly interacting with their antigens, thus leading to multi-system damage of SLE (29, 30). Besides, B cells can contribute to SLE pathogenesis through additional pathways. For example, B cells can work as antigen presenting cells and correlate with activation of other crucial lymphocytes in SLE; Certain subtype of B cells may secrete anti-inflammatory cytokines in SLE. Therefore, the study of B lymphocytes can potentially unravel important pathogenic mechanisms of SLE. Previous studies have delineated several signaling pathways that contributed to the over-reactivity of B cells in SLE, including Janus kinase/signal transducer and activator of transcription (JAK-STAT), B cell receptor/phosphatidylinositol 3-kinase (PI3K)/protein kinase B (AKT), and TLRs (31), although the detailed molecular mechanisms remain to be elucidated. Mice that are deficient in various inhibitory molecules that dampen B-cell receptor (BCR) signaling, such as SHIP-1 (32), Lck/Yes novel tyrosine kinase (LYN) (33), or Fcγ receptor IIb (FcγRIIb) (34), develop systemic autoimmunity. Studies of the aberrant expression of several miRNAs in the B cells of SLE patients have reported that miR-7, miR-155, miR-30a, and miR-15a were up-regulated, while miR-1246 were down-regulated (**Figure 3**), and researches have preliminarily demonstrated their role in SLE.

A recent study reported that he up-regulation of miR-7 in the B cells of SLE patients can negatively regulate the expression of phosphatase and tensin homolog (PTEN), which results in up-regulated activation of PI3K/AKT signaling (35, 36). MiR-7-mediated down-regulation of PTEN/AKT signaling promoted the differentiation of B cells into plasmablasts/plasma cells and formation of spontaneous germinal center in a MRL^lpr/lpr^ mouse model (37). The treatment of miR-7 antagomir showed its therapeutic value *in vivo* in MRL^lpr/lpr^ mice, which consequently alleviated the clinical manifestations of organ damage in lupus mice model (37). Thai et al. (38) reported that ablation of miR-155 in lupus-prone mice with death receptor deficiency (Fas^lpr^) reversed the reduced expression of SHIP-1 to normal levels, which acted as downstream of inhibitory cell-surface receptors, such as FcγRIIb. Subsequently, these processes contributed to decreasing serum levels of IgG anti-dsDNA antibodies and kidney inflammation, and then reduced autoantibody responses in lupus-like diseases. In addition, miR-155 acts as a suppressor of autoimmunity through transcriptional repression of PU.1 (a crucial regulator of B-cell development) and TNF-α, which in turn suppresses B cell-activating factor belonging to the TNF family and CD19 protein expression (39). Like miR-7 and miR-155, Liu et al. (40) observed that miR-30a expression was significantly increased in the B cells of SLE patients and miR-30a directly decreased expression of LYN by targeting the 3’-UTR of LYN mRNA. LYN is a member of the Src family of non-receptor tyrosine kinases (41) and a key mediator in several pathways of B cell activation, such as CD19 and CD180 (33). In addition, significantly decreased LYN levels have been observed in the B cells of SLE patients (42). Thus, high miR-30a expression can regulate B cell proliferation and antibody production in SLE patients, suggesting that miR-30a might be involved in pathogenesis of SLE. B cell lymphoma-2 (Bcl-2) is an important component of the apoptotic pathway. In the human genome, four members of the miR15/16 family share the same 9-bp Bcl-2-complementarity sequence. This functional redundancy indicates that Bcl-2 expression is regulated by a very fine mechanism. MiR-15a has been demonstrated to potentially regulate the balance of B-10 and B-1 cell subsets and was positively correlated to autoantibody levels in lupus due to differential expression in B cell subpopulations (43). Down-regulation of Bcl-2 expression by miR-15a overexpression activates the apoptotic pathway of the B-10 subset (44), which has been shown to suppress lupus in a B/W mouse model and other inflammatory diseases *via* preferential production of IL-10 (45). The induced loss of this regulatory B cell subset may lead to more prominent autoantibody production (46).

On the contrary, miR-1246 expression was negatively correlated with the activation of B cells in SLE patients (47). Further research verified that decreased miR-1246 expression reduced the inhibitory effect on the expression of early B cell factor 1 (EBF1), which contributed to the development, activation, and proliferation of B cells *via* activation of the AKT signaling pathway. The upregulated expression of EBF1 increases the production of the B cell surface co-stimulatory molecules CD40, CD80, and CD86, which then enhances B cell function.

As listed above, the underlying mechanism of miRNA-7 or miRNA-155 contribute to B cell hyperresponsiveness and autoantibody responses have been well studied. Further, single intervention with miRNA-7 or miRNA-155 alleviate the disease manifestation or inhibits lupus development in mice model suggesting that their critical role in lupus progression and their potential as treatment strategy in SLE. The miRNA-15a, miRNA-30a and miRNA-1246 are demonstrated to be involved in certain factors that contribute to B cells overactivation. Further studies are needed to show the role of miRNAs in SLE and whether it has the potential to be developed into new therapeutic targets.

## The Roles of miRNAs in Aberrant Activation of CD4^+^ T Cells in SLE

The critical role of T cells in the pathogenesis of SLE has been confirmed by initiating and amplifying the inflammatory process through directly contacting with other immune cells in lymphoid organs, secreting pro-inflammatory cytokines or mediating direct effects on target tissues. Naive CD4^+^ T cells can differentiate into various Teff cell subsets, including Th1, Th17, Th2 and follicular helper T (Tfh) cells. Continuously stimulated T cells in lupus are likely to contribute to the disease by secreting inflammatory cytokines and supporting B cells to produce a wide variety of high affinity autoantibodies through contact-dependent mechanisms (mediated by CD40L:CD40, OX40L:OX40, and so on), which is an important characteristic of SLE. In addition, stimulation of autoreactive CD4^+^ T cells can foster the differentiation of CD8^+^ T cells into cytotoxic T lymphocytes along with the employment of inflammatory cytokines. However, the mechanism that causes the aberrant activation, differentiation and function of T cells in SLE remains largely unclear.

Genome-wide analysis has revealed that global DNA methylation levels are reduced by 15%–20% in the CD4^+^ T cells of patients with active SLE (48), especially genes involved in disease pathogenesis and progression, such as ITGAL, CD40LG, CD70, and PPP2CA. DNA methylation is an elementary determinant of the chromatin structure that is established during development by *de novo* DNA methyltransferases (DNMTs) with potent suppressive effects on transcription. DNMT1 serves to maintain the methylation status of proliferating cells (49). Moreover, the T cells of mice treated with procainamide and other inhibitors of DNA methylation can induce SLE in recipient mice (50). The pathological significance of the autoreactivity induced by inhibiting DNA methylation in T cells was further investigated. Pan et al. (51) demonstrated increased expression of miR-21 and miR-148a in SLE patients and SLE-prone MRL/lpr mice, and that both miRNAs reduced DNMT1 expression, which contributed to epigenetic changes *via* DNA hypomethylation. MiR-21 indirectly inhibits DNMT1 expression by targeting the important autoimmune gene RAS guanyl nucleotide-releasing protein 1, which is a critical regulator of the upstream RAS/mitogen-activated protein kinase pathway signaling cascade of DNMT1 in T cells (51). Another study confirmed that enhanced miR-21 expression also contributed to the aberrant phenotype of T cells in SLE which could be through interaction with its predicted target gene, programmed cell death protein 4 (52). Silencing of miRNA-21 *in vivo* can efficiently alter the course of autoimmune splenomegaly in lupus mice (53). On the other hand, miR-148a directly inhibits DNMT1 expression by targeting the protein coding region of the transcript. These data clearly showed that abnormally expressed miRNAs in SLE patients had a critical functional link with the aberrant DNA hypomethylation in lupus CD4^+^ T cells, resulting in the overexpression of autoimmune-associated methylation-sensitive genes, such as those that encode CD70 (tumor necrosis factor (ligand) superfamily, member 7 [TNFSF7]), CD40 ligand (TNFSF5), and lymphocyte function-associated antigen 1 (LAF-1, integrin αLβ2, CD11a/CD18) (54), which contributed to the autoreactivity and overstimulation of CD4^+^ T cells in SLE (51). Many studies have found that expression of miRNAs, such as miR-126 (55) and miR-29b (56), was markedly altered in the CD4^+^ T cells of SLE patients and was involved, either directly or indirectly, in decreasing DNA methylation levels, which led to aberrant activation and differentiation of CD4^+^ T cells.

In addition to aberrant DNA methylation, miRNAs in the CD4^+^ T cells of patients with SLE can regulate T cell activation in other ways. A recent study found that miR-142-3p/5p expression was decreased in the CD4^+^ T cells of patients with SLE (57). MiR-142-3p specifically targets members of the signaling lymphocytic activation molecule (SLAM) family, including interleukin (IL-10) and CD84, while miR-142-5p targets the 3’-UTR of SLAM-associated protein (SAP). Thus, decreased miR-142-3p/5p expression contributes to the up-regulation of CD84 and IL-10/SAP, resulting in increased T cell function and immunoglobulin (Ig) G production in co-cultured B cells. Reduced expression of miR-142-3p/5p in the CD4^+^ T cells of patients with SLE activates T cells and hyperstimulates B cells (58). Besides, overexpression of signal transducer and activator of transcription 1 (STAT1) and under-expression of apoptosis inhibitory protein 5 (API5) in the T cells of SLE patients are targeted by miR-145 and miR-224, respectively. Thus, aberrant expression of miR-145 and miR-224 can promote T cell activation-induced cellular apoptosis by suppressing API5 expression and SLE-associated nephritis by enhancing STAT1 expression (59) (**Figure 2B**).

According to current studies, a critical functional link between miRNAs and the lupus CD4^+^ T cells have been connected by the potential interplay between miRNAs and critical molecules such as SLAM family, STAT-1, DNMT1 which contribute to T cells abnormalities and hypomethylation in SLE. Moreover, transfection of miRNAs or miRNA inhibitors have beneficial effects on alleviating CD4^+^T cell disease phenotype, highlighting the important role of miRNAs in lupus-like CD4^+^T phenotype transformation. Further researches are needed to evaluate the place of miRNAs in the complicated regulatory networks of DNA hypomethylation in SLE.

## The Roles of miRNAs in Breakdown of Immune Tolerance in SLE

SLE is characterized by a wide array of immune tolerance breakdown with systemic inflammation involving the dysregulation of immune responses. Tregs are a unique subpopulation of CD4^+^ T cells with an indispensable role in maintaining self-tolerance by suppressing autoreactive lymphocytes and suppressing excessive immune responses by controlling the responses of Teffs (60, 61). Tregs characteristically express CD25 (IL-2 receptor α chain) and the lineage-specific transcription factor forkhead box P3 (Foxp3). An imbalance between effector T cells (Teffs) and regulatory T cells (Tregs) is central to the pathogenesis of SLE. Pathogenic Teffs in SLE are mainly Tfh and Th17 cells. Tfh cells assist the activation of B cells to produce autoantibodies, which results in multiple organ damage. Th17 cells secrete pro-inflammatory cytokine that amplify immuno-inflammatory responses, resulting in tissue damage. Previous studies have reported that the proportions of Tfh and Th17 cells were increased in SLE patients and correlated with disease severity (62), although the underlying mechanisms remain unclear.

MiR-125a is commonly downregulated in the peripheral CD4^+^ T cells of patients with various autoimmune diseases, such as SLE and Crohn’s disease, which suppresses several factors of Teffs, including STAT3, IFN-γ, and IL-13 (63). A recent study reported consistent delivery of miR-125a into splenic T cells in a mouse model of SLE with the use of a nano-delivery system, which significantly alleviated disease progression by reversing the imbalance of Teffs and Tregs (64). These findings point to miR-125a as a critical factor to restrict the development of SLE by stabilizing Treg-mediated immune homeostasis. Xie et al. (65) reported that miR-34a in the peripheral blood mononuclear cells (PBMCs) of SLE patients played a potential role in disease activity and expression levels were positively correlated with several serum disease indexes, including rheumatoid factor, antistreptolysin antibody, erythrocyte sedimentation rate, and C-reactive protein. Further research demonstrated that miR-34a attenuated human and murine Foxp3 expression (66, 67) by targeting the 3ʹ-UTR, and then limited the differentiation of Tregs, which impaired the balance of Tregs and Th17 cells. The release of IL-6 or tumor necrosis factor (TNF)-α in the inflammatory environment can activate the NF-κB pathway and increase the expression levels of miR-34a by enhancing promoter activity, resulting in Foxp3 downregulation and Treg/Th17 imbalance (65). Meanwhile, miR-31 overexpression also inhibits differentiation of Tregs by targeting Foxp3 and other molecules that are indispensable for the development of Tregs, such as G protein-coupled receptor class C group 5 member A and protein phosphatase 6c (68, 69). MiR-142-5p positively regulates intracellular levels of cyclic adenosine monophosphate (cAMP) to maintain the suppressive function of Tregs (58). In contrast, miR-142-3p can restrict cAMP levels in CD4^+^ T cells, which compromises the inhibitory function of Tregs (70). Many studies have confirmed that the expression levels of the abovementioned miRNAs were markedly altered in the Tregs of SLE patients and aberrant regulation of Tregs was involved in the development of SLE. Other miRNAs, such as miR-99a, miR-17 (71, 72), and miR150 (73), also have the ability to regulate the function of Tregs either directly or indirectly according to researches, which potentially provides new clues for future research on the development of SLE (**Figure 2C**).

In recent years, other cells with regulatory capabilities were revealed such as B regulatory cells and NK cells. As we mentioned above, miR-15a overexpression contributes to activating the apoptotic pathway of the B-10 subset (44) and then weaken its suppress effects on SLE and other inflammatory diseases (45). In another study, the recognition between iNKT cells and B cells through CD1d is associated with the tolerance of NKT cells (74). The increased miR-155 contribute to inhibiting CD1d expression in B cells by directly targeting the 3’-UTR of CD1d, and thus impair the tolerance of NKT cells (75).

## The Roles of miRNAs in Abnormally Increased Production of Inflammatory Cytokines in SLE

Cytokines are a family of small proteins that play crucial roles as messengers of immune pathways and in the regulation of leukocyte activation. It is thought that the balance between proinflammatory and anti-inflammatory cytokines influences the clinical manifestations in many inflammatory diseases such as SLE and rheumatoid arthritis. These cytokines are mainly produced by helper T (Th) cells, which can be classified based on functional effects into T helper (Th) 1 (IFN-γ, IL-2, TNF-α), Th2 (IL-4, IL-5 and IL-6), Th17 (IL-17), and Tregs (IL-10). High levels of inflammatory cytokines may lead to the exacerbation of inflammatory responses, apoptosis, and production of autoantibodies that initiate and sustain SLE disease activity (76, 77). Dysregulation of chemokine production has been linked to the clinical manifestations and disease activity of SLE (78). Multifactorial components contribute to the immune modulation of cytokines, such as genetic polymorphisms, environmental factors, and hormonal alterations, among others, leading to irreversible impairment of self-immunological tolerance.

Type I IFNs are a family of cytokines produced by innate immune cells, especially plasmacytoid DCs and tissue cells, when viral components are perceived *via* retinoic acid-inducible-like receptors and TLRs. Increasing serum levels of IFN in lupus patients was described more than 40 years ago (79). Among the key immunological alterations in SLE, type I IFNs and related signaling pathways have been shown to play pivotal roles in disease pathogenesis (80, 81). Overexpression of type I IFNs can cease tolerance and induce autoimmune diseases *via* increased expression of major histocompatibility complex I (MHC I) molecules (82, 83), which enhances the cross-presentation of exogenous antigens. Likewise, the expression of other molecules related to the immune response promoted by IFN include MHC II, CD40, CD80, and CD86 in addition to chemokines and related cognate receptors, such as chemokine (C-X-C motif) ligand 10 and CXC chemokine receptor 3 (84). In response to IFN stimulation, DCs mature and transform into active antigen-presenting cells (APCs). Potent APCs induce the differentiation of naive CD4^+^ T cells, promote the development of CD8^+^ memory T cells and differentiation of Teffs, and suppress the functions of Tregs, which can collectively lead to the expansion of autoreactive T cells (85). The function of B cells is also impacted by type I IFNs, which is specifically manifested by extended survival and activation, leading to enhanced antibody production (86), ultimately resulting in the development of autoimmune diseases.

Under normal physiological conditions, immune cells spontaneously and negatively regulate TLR signaling through various mechanisms, so as to avoid abnormal activation and to maintain immunological balance (87). Extracellular miRNAs were shown to act as cell-to-cell regulators through a nonconventional mechanism of scilicet interactions with innate immune RNA receptors, such as TLR 7 and 8 (88, 89). MiR-146a is a negative regulator of TLR signaling (90), which can be induced by various stimuli, such as lipopolysaccharides, imiquimod R837, type A CpG oligonucleotides, and type I IFNs (51). Hou et al. (91) used bioinformatics tools to demonstrate that mature miR-146a reduced expression of multiple components in the type I IFN signaling cascades, including interleukin-1 receptor-associated kinase 1, tumor necrosis factor receptor-associated factor 6, IFN regulatory factor 5, and STAT1, thereby directly attenuating downstream activation of type I IFNs. Therefore, it appears that miR-146a deficiency is a causal factor that contributes to abnormal activation of the type I IFN pathway in SLE. Moreover, the coordinated activation of the type I IFN pathway was notably reduced after the introduction of miR-146a into the PBMCs of SLE patients. Collectively, these results suggested that exogeneous regulation of miR-146a is a promising therapeutic strategy for SLE. In 2010, Wang et al. (92) reported a positive association between miR-155 and IFN-α. MiR-155 feedback induced by viral infection promotes type I IFN signaling by targeting suppressor of cytokine signaling 1, a canonical negative regulator of type I IFN signaling, and mediates the enhancing effect of miR-155 on type I IFN-mediated antiviral responses. However, further studies are needed to clarify the role of miR-155 in type I IFN signaling and to investigate any possible correlations with SLE. Otherwise, the results of a luciferase reporter assay using Rat-1 fibroblasts stably expressing miR-181b revealed that miR-181b directly and negatively regulated IFN-α (93).

When regulated upon activation, normal T cell express and secrete CCL5 (RANTES) which is a key chemokine for T cell recruitment to inflammatory tissues, and active expression is known to enhance levels and detrimental effects of inflammatory factors in arthritis, nephritis, and a myriad of other inflammatory disorders (94). Downregulated expression of miR-125a in SLE patients contributes to blunting the negative regulation of RANTES expression by targeting Kruppel-like factor 13 in active T cells. Hence, miR-125a could potentially serve as a therapeutic target for the treatment of SLE *via* regulation of inflammatory chemokine production (95).

## Conclusions and Perspectives

**Figure 4:**
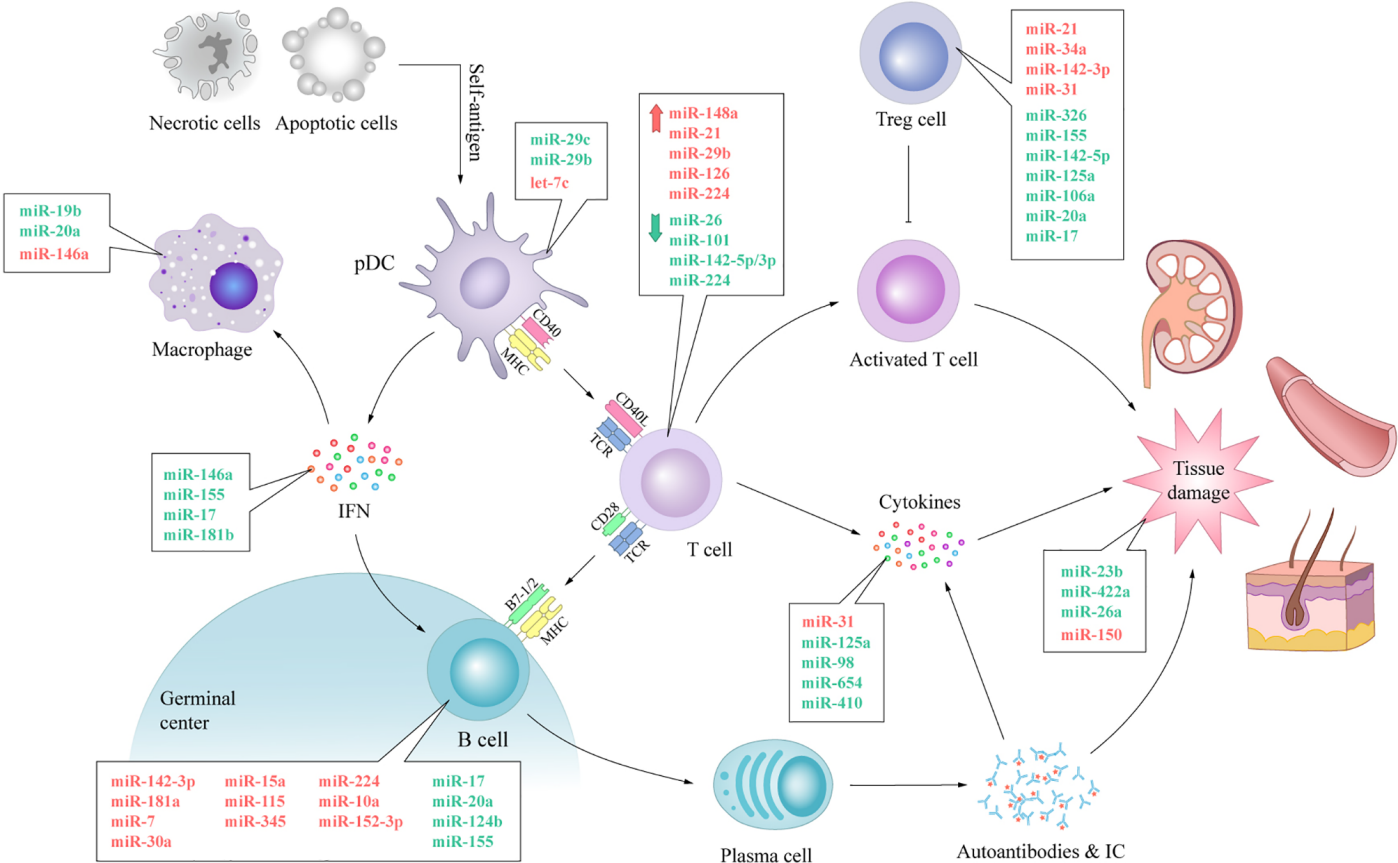
The expression of miRNAs and their involvement in immunological dysfunction of SLE. The aberrant expression of miRNAs can be noticed in almost every vital process of SLE. The expression levels of anti-inflammatory miRNAs, such as miR-19b, miR-146a, miR-142-5p, miR-124b and miR-422a, decrease, while the expression levels of pro-inflammatory miRNAs, such as miR-146a, miR-224, miR-29b, miR-31 and miR-150, increase. Dysregulated miRNAs disturb the normal biological procedure of immune response by swaying the expression of pivotal protein molecule (e.g. CD40, CD40L, IFN, BAFF) directly or indirectly. These processes lead to autoimmune and tissue damage with aberrantly activated T lymphocytes, over-activated B cells, autoantibody accumulation and abnormally increased inflammatory cytokines in SLE. IC, immune complex; TCR, T cell receptor.

SLE is a heterogeneous chronic inflammatory autoimmune disorder, which is characterized by aberrant activation of lymphocytes, auto-antibodies, and inflammatory cytokine production (1). The highly heterogeneous nature of SLE has hampered a comprehensive understanding of the etiology of the disease in terms of both the underlying pathogenic processes and manifestations. Although researchers have spent a great deal of effort to figure it out, it is clear that there is still much to understand regarding the intricate network of SLE. MiRNAs are a family of small noncoding RNA molecules that provide quantitative regulation of genes at the post-transcriptional level by targeting mRNA translation or degradation (3). This summary of the current research results has shown that various, ubiquitous and functional miRNAs are involved in most immunological events leading to SLE. The upregulation of let-7c contributes to proinflammatory features of DCs in SLE (23); MiR-7 is associated with the overactivation of B cells and subsequent autoantibody production (37); MiR-21 up-regulation contributes to T cell hyperactivity (51); MiR-34a is associated with defection of Tregs and immune tolerance breakdown (65); Transfection of miR-98 alleviates the increased production of inflammatory cytokines (96). Different miRNAs can target the same mRNA, and one miRNA can target multiple mRNAs. **Table 1** and **Figure 4** list miRNAs that are preliminarily reported as being affiliated with immunoregulation in SLE. More extensive and in-depth studies on miRNAs are ongoing and the miRNAs involved in the pathogenesis of SLE are not limited to those described here.

**Table 1:** The roles of miRNAs in the pathogenesis of SLE.

Events	miRNA	Levels	Mechanism	Biological function	Experimental subject	Reference
DCs	let-7c	↑	suppresses SOCS1 expression	contributes to a broad spectrum of proinflammatory DC phenotype	DCBlimp1^ko^ mice	(23)
	miR-155	↑	enhances TLR7-induced CD40 expression	contributes to the hyperactivated TLR7 response in lupus pDCs	Lupus-Prone NZB/W F1 mice	(25)
	miR-150	↓	inhibits the expression of TREM-1	enhances the inflammation responses in splenic cDCs in lupus prone mice	MRLlpr/lpr mice and C57BL/6 wild type mice	(26)
B cell	miR-1246	↓	reduces the inhibitory effect on the expression of EBF1 and activates AKT signaling pathway	contributes to the development, activation, and proliferation of B cells	PBMCs from SLE patients and healthy controls	(47)
	miR-15a	↑	targets important genes involving cell cycle (e.g. cyclin D1) or cell apoptosis (e.g. Bcl-2)	disrupts the balance between regulatory B cells (B-10) and the pathogenic B cells (B-2) and increases the anti-dsDNA autoantibody levels	SLE mouse model	(46)
	miR-30a	↑	decreases the expression of Lyn such as CD19 and CD180	regulates the B cell proliferation and antibody production	PBMCs from SLE patients and healthy controls	(40)
	hsa-miR-345	↑	inhibits IRF8	regulates the differentiation of B cells	PBMCs from SLE patients and healthy controls	(97)
	hsa-miR-224	↑	contributes to IRF4 overexpression and increass production of IL-21	B cell hyperresponsiveness		
	hsa-miR-10a	↑	inhibits IL-8 during the inactive phase	blocks the generation of autoreactive antibodies by B lymphocytes		
	miR-155	↑	contributes to the reduction of SHIP-1 expression partly	increases serum IgG anti-dsDNA antibodies and kidney inflammation	Fas^lpr^ lupus-prone mice and their PBMCs	(38)
			increases the expression of PU.1 and TNF-α, which in turn promotes BAFF and CD19 protein expression	disrupts peripheral tolerance in B cells and thereby induces autoantibody production increase the SLEDAI-2K	PBMCs from SLE patients and healthy controls	(39)
	miR-152-3p	↑	increases the BAFF expression by targeting and decreases the expression of KLF5	causes B-cell self-reactivity and autoantibody production	PBMCs from SLE patients and healthy controls	(98)
	miR-7	↑	negatively regulates the PTEN expression and down-regulates activation of PI3K/AKT signaling	promotes B cell differentiation into plasmablasts/plasma cells and spontaneous germinal center (GC) formation	MRL lpr/lpr lupus mice and their PBMCs	(35–37)
T cell	miR-21	↑	targets RASGRP1 to diminish Ras-MAPK pathway signaling and inhibit DNMT1 expression	contributes to DNA hypomethylation and T cell hyperactivity	SLE patients, lupus-prone MRL/lpr mice and healthy human PBMCs	(51–53)
			decreases expression level of PDCD4 to regulate IL-10 and CD40L	contributes to aberrant T cell phenotype	PBMCs from SLE patients and healthy controls	
	miR-148a	↑	targets DNMT1 to regulate CD70 and LFA-1	contributes to DNA hypomethylation and T cell hyperactivity	SLE patients, lupus-prone MRL/lpr mice and healthy human PBMCs	(51)
	miR-126	↑	targets DNMT1 to induce demethylation and up-regulate CD70 and CD11a	contributes to DNA hypomethylation and T cell hyperactivity	PBMCs from SLE patients and healthy controls	(55, 99)
	miR-29b	↑	targets Sp1 to negatively regulate DNMT1 and increases the expression of CD70 and CD11a gene	contributes to DNA hypomethylation and T cell hyperactivity	PBMCs from SLE patients and healthy controls	(56)
	miR-145	↓	targets STAT-1 mRNA and ehances its expression level	associates with lupus nephritis significantly	PBMCs from SLE patients and healthy controls	(59)
	miR-224	↑	targets API5 mRNA and decreases its expression level	accelerates T cell activation-induced cell death in Jurkat cells		
	miR-142-5p	↓	targets SLAM associated protein (SAP)	T cell activation and B cell hyperresponsiveness	PBMCs from SLE patients and healthy controls	(57)
	miR-142-3p	↓	targets SLAM family (IL-10 and CD84)	T cell activation and B cell hyperresponsiveness		
immune tolerance	miR-21	↑	regulates FOXP3 expression positively	negatively regulates Treg cell development	umbilical cord blood mononuclear cells (UCBMC)	(68)
	miR-142-3p	↑	reduces the level of intracellular cAMP by inhibiting AC9 production	limits the suppressor function of Treg cells	splenocytes from naive BALB/c mice	(70)
	miR-31	↑	targets Gprc5a, Ppp6C and Foxp3	negatively regulates Treg cell generation and differentiation	PBMCs from murine and SLE patient	(68, 69)
	miR-17	↓	targets TGFβRII and CREB1	inhibits iTreg differentiation and facilitates effector T-cell responses	mice and PBMCs from SLE patients	(71, 72)
	miR-142-5p	↓	enhances Pde3b transcription and reduces the level of intracellular cAMP	key determinant of Treg function and peripheral immune tolerance	spleen and peripheral lymph nodes cells from mice	(58)
	miR-34a	↑	targets and attenuates the expression of Foxp3 gene	inhibits Treg cell differentiation and disrupts Treg/Th17 balance	PBMCs from murine and SLE patient	(65–67)
	miR-125a	↓	insufficiently targets and increases the expression of STAT3, Ifng, Il13	labilizes the immunoregulatory capacity of Treg cells	mice, SLE patient and their PBMCs	(63, 64)
	miR-15a	↑	activating the apoptotic pathway of the B-10 subset	weaken suppress effects of B-10 cells on SLE and other inflammatory diseases	Female (NZB × NZW)F1 or B/W mice	(46)
	miR-155	↑	inhibiting CD1d expression in B cells	impair the tolerance of NKT cells	Female C57BL/6J and MRL/lpr lupus-prone mice	(75)
inflammatory chemokine	miR-146a	↓	impairs negative regulation of multiple components expression in the type I IFN pathway, including IRAK1, TRAF6, IRF-5 and STAT-1	directly activates downstream of type I IFN	C57BL/6 mice, HEK293 cells	(90, 91)
	miR-125a	↓	increases KLF13 gene expression and hence RANTES expression	regulates inflammatory chemokines production and contribute to organ inflammation	PBMCs from SLE patients and healthy controls	(95)
	miR-181b	↓	directly regulates AID and IFN-α mRNAs	impairs negative regulation to IFN-α	PBMCs from SLE patients and healthy controls	(93)
	miR-410	↓	impairs negative regulation to transcription activity of STAT3	increases the expression levels of IL-10	PBLs from SLE patients and healthy controls	(100)
	miR-302d	↓	increases IRF9 gene expression and enhances ISG expression	negatively correlates with IFN score	PBMCs from SLE patients and healthy controls	(101)
	miR-31	↑	targets serine/threonine kinase 40	promotes NF-κB signaling to enhance inflammatory cytokine production.	PBMCs and splenocytes in MRL/lpr mice	(102)
	miR-98	↓	impairs targeting gene IL-6	promotes STAT3-mediated cell proliferation and inflammatory cytokine production	PBMCs from SLE patients and healthy controls	(96)
	miR-654	↓	inhibits MIF expression by binding to MIF mRNA	selectively suppresses the phosphorylation of ERK and AKT, reduces downstream inflammatory cytokine production	PBMCs from SLE patients and healthy controls	(103)

Based on the results of current studies, the exact relationship between miRNAs and the pathogenesis of SLE cannot be concluded arbitrarily. Although dysregulation of miRNAs has been reported to be involved in most important events in the progression of SLE, there is no conclusive evidence that abnormal miRNA expression is a cause or merely a consequence of SLE. Many of current studies are limited to the analysis of how miRNA affects SLE at the cytological level. These studies demonstrated that abnormally expressed miRNAs in PBMCs could lead to lupus-like cellular phenotypes characterized by overexpression of TLRs (25, 57, 104) or co-stimulatory molecules (51, 73), enhanced cell signal transduction pathways (23, 59, 65, 69, 70), increased inflammatory cytokines (66, 67, 91), which have been found in abundance in SLE. However, whether small miRNAs can be a cause for the development of this intricate disease in healthy individuals has not been demonstrated in the present study. Even so, the role of miRNAs in molecular regulatory network of SLE is fascinating and have suggested an exciting avenue to enrich our understanding of SLE. Investigations into miRNAs and related molecular mechanisms involved in SLE will help to clarify the pathogenesis of this complex disease and potentially facilitate the identification of new treatment modalities. MiRNA-based therapeutic agents have been developing for the treatment of a variety of diseases (15, 105). Gene knockout and transfection of several miRNAs have been demonstrated to alleviate disease activity of SLE in mice (23, 38). In addition, small-molecule drugs that target the biogenesis of miRNA-155 for the treatment of SLE have been recently discovered (106). However, these approaches have not yet reached the level of clinical application. Current studies have made significant progress to analysis how miRNA affects SLE at the cytological level, but there are still many efforts to be invested in how far miRNAs may have played a role and whether it has the potential to be developed into new therapeutic targets. Thus, it is still too early to make conclusions about the therapeutic effect of miRNAs on lupus. Despite the considerable therapeutic potential, the results of basic studies are still a long way from being translating to clinical care, thus further research in this challenging field is urgently needed.

## Author Contributions 

MC, KM, and LS were involved in the conception of the study. MC and KM were involved in writing the article. MQ, ZH, ZD, YL, XL, QZ and CL critically revised the manuscript. All authors contributed to the article and approved the submitted version.

## Funding

The work was supported by Foundation of Popularization project Department of Sichuan Health commission (19PJYY0731), Foundation of “apricot grove scholar” of Chengdu University of Traditional Chinese Medicine (2019yky10), Xinlin scholars Science Foundation of Chengdu University of Traditional Chinese Medicine (ARQN2019007), Science and technology project fund for Returned Students of Sichuan Province (00809504), The project of 2020 High-level Overseas Chinese Talent Returning Funding.

## Conflict of Interest

The authors declare that the research was conducted in the absence of any commercial or financial relationships that could be construed as a potential conflict of interest.

## Publisher’s Note

All claims expressed in this article are solely those of the authors and do not necessarily represent those of their affiliated organizations, or those of the publisher, the editors and the reviewers. Any product that may be evaluated in this article, or claim that may be made by its manufacturer, is not guaranteed or endorsed by the publisher.

## Glossary

**Table:**

SLE	Systemic lupus erythematosus
miRNAs	microRNAs
auto-Abs	autoantibodies
DCs	dendritic cells
TLR	Toll-like receptor
NF-κB	nuclear factor kappa-light-chain-enhancer of activated B cells
pri-miRNAs	primary RNA transcripts
RNase III enzyme	ribonuclease III enzyme
pre-miRNA	precursor miRNAs
RISC	RNA-induced silencing complex
mRNA	messenger RNA
3’-UTR	3’-untranslated region
mDC	myeloid DC
pDC	plasmacytoid DC
Blimp1	B lymphocyte–induced maturation protein-1
SOCS	suppressor of cytokine signaling
SHIP-1	SH2 domain-containing inositol 5’-phosphatase 1
JAK-STAT	Janus kinase/signal transducer and activator of transcription
PI3K	phosphatidylinositol 3-kinase
AKT	protein kinase B
BCR	B cell receptor
LYN	Lck/Yes novel tyrosine kinase
FcγRIIb	Fcγ receptor IIb
PTEN	phosphatase and tensin homolog
Bcl-2	B cell lymphoma-2
EBF1	expression of Early B cell factor 1
Tfh cells	follicular helper T cells
DNMTs	DNA methyltransferases
RASGRP1	rasguanyl-nucleotide-releasingprotein1
TNFSF	tumour necrosis factor superfamily
LAF-1	lymphocyte function-associated antigen 1
SLAM	signaling lymphocytic activation molecule
IL-10	interleukin 10
SAP	SLAM associated protein
IgG	immunoglobulin G
STAT-1	activator of transcription-1
API5	apoptosis inhibitory protein 5
Foxp3	forkhead box P3
Teff cell	effector T cell
Treg cells	regulatory T cells
PBMCs	peripheral blood mononuclear cells
TNF α	tumour necrosis factor α
cAMP	cyclic adenosine monophosphate
Th cell	T helper cell
IFN-1	type 1 interferon
MHC I	major histocompatibility complex I
CXCL	chemokine (C-X-C motif) ligand
CXCR 3	CXC chemokine receptors 3
APCs	antigen-presenting cells
RANTES	regulated on activation, normal T cell expressed and secreted
